# Transcriptional Regulation of Methanol Dehydrogenases in the Methanotrophic Bacterium *Methylococcus capsulatus* Bath by Soluble and Insoluble Lanthanides

**DOI:** 10.1264/jsme2.ME23065

**Published:** 2023-12-12

**Authors:** Ruoyun Xie, Motoko Takashino, Kensuke Igarashi, Wataru Kitagawa, Souichiro Kato

**Affiliations:** 1 Division of Applied Bioscience, Graduate School of Agriculture, Hokkaido University, Kita-9 Nishi-9, Kita-ku, Sapporo 060–8589, Japan; 2 Bioproduction Research Institute, National Institute of Advanced Industrial Science and Technology, 2–17–2–1 Tsukisamu-Higashi, Toyohira-ku, Sapporo 062–8517, Japan

**Keywords:** lanthanides, methanotroph, methanol dehydrogenase, gene expression, chelator compounds

## Abstract

The effects of soluble and insoluble lanthanides on gene expression in *Methylococcus capsulatus* Bath were investigated. Genes for lanthanide-containing methanol dehydrogenases (XoxF-MDHs) and their calcium-containing counterparts (MxaFI-MDHs) were up- and down-regulated, respectively, by supplementation with soluble lanthanide chlorides, indicating that *M. capsulatus* has the “lanthanide switch” observed in other methanotrophs. Insoluble lanthanide oxides also induced the lanthanide switch and were dissolved by the spent medium of *M. capsulatus*, suggesting the presence of lanthanide-chelating compounds. A transcriptome ana­lysis indicated that a gene cluster for the synthesis of an enterobactin-like metal chelator contributed to the dissolution of insoluble lanthanides.

Since methane is the second most important greenhouse gas after carbon dioxide, a more detailed understanding of its generation and consumption in natural environments is important. Methane-oxidizing bacteria (methanotrophs) oxidize methane aerobically, which significantly contributes to mitigating the release of methane produced in anaerobic environments (Hanson and Hanson, 1996). Therefore, the mechanisms by which environmental factors, such as the availability of trace elements, affect the activities of methanotrophs warrant further study (Semrau *et al.*, 2010, 2018).

Rare earth elements (REEs) form a chemically uniform group that includes 15 lanthanides (lanthanum [_57_La] to lutetium [_71_Lu]) and two non-lanthanides (scandium [_21_Sc] and yttrium [_39_Y]). REEs are not rare in terms of their abundance. Their contents in the Earth’s crust, soil, and sediment particles are as high as 200 ppm, which are similar to those of general metals, such as Cu and Zn (Tyler, 2004). Although REEs were originally considered to be non-essential elements for life, specific pyrroloquinoline quinone (PQQ)-dependent methanol dehydrogenases (XoxF-MDHs) were found to contain lanthanides as their cofactor, whereas their well-characterized counterparts (MxaFI-MDHs) were Ca^2+^-containing enzymes (Hibi *et al.*, 2011; Chistoserdova, 2016). Most methanotrophs have both MxaFI- and XoxF-MDHs, while others only have XoxF-MDHs (Beck *et al.*, 2013; Pol *et al.*, 2014; Kato *et al.*, 2020). In some methylo- and methanotrophs, genes for XoxF and MxaFI were shown to be up- and down-regulated, respectively, in the presence of lanthanide, known as the “lanthanide switch” (Farhan Ul Haque *et al.*, 2015; Vu *et al.*, 2016; Gu and Semrau, 2017; Krause *et al.*, 2017; Daumann *et al.*, 2022). The lanthanide switch has also been reported in non-methylotrophic bacteria with two types of PQQ-dependent alcohol dehydrogenases (Wehrmann *et al.*, 2017). The mole­cular mechanisms underlying the lanthanide switch have been characterized in some methylotrophs (Chu *et al.*, 2016; Ochsner *et al.*, 2019; Roszczenko-Jasińska *et al.*, 2020) and methanotrophs (Groom *et al.*, 2019; Shiina *et al.*, 2023). These studies revealed that TonB-dependent receptors and the ABC transport system contributed to the sensing and uptake of lanthanides.

Previous studies on the bacterial utilization of REEs generally used REE chloride salts, which are easily soluble and readily available. However, REEs occur in nature in the form of insoluble oxides. Dissolved REEs are generally present at sub-nanomolar concentrations in freshwater and seawater (Elderfield *et al.*, 1990; Shiller *et al.*, 2017), which is three to four orders of magnitude lower than the minimum requirement for methylo- and methanotrophs (generally the sub-micromolar order) (Chu *et al.*, 2016; Gu and Semrau, 2017). Bacteria utilizing lanthanides are considered to solubilize lanthanide oxides, possibly through similar mechanisms that make insoluble Fe and Cu available. Many bacteria are known to produce metal-chelating compounds, namely, siderophores (Fe chelators) and methanobactins (Cu chelators), in order to solubilize insoluble Fe and Cu, respectively (Ahmed and Holmström, 2014; Semrau *et al.*, 2018). Juma *et al.* (2022) recently reported that the methylotrophic bacterium *Methylobacterium aquaticum* strain 22A secreted small chemicals that dissolved insoluble lanthanum oxide (La_2_O_3_), and the gene cluster for the biosynthesis of staphyloferrin B-like siderophores was necessary for its growth on La_2_O_3_, indicating that metal-chelating compounds play critical roles in the utilization of insoluble lanthanides. However, the utilization of insoluble lanthanides and the underlying mole­cular mechanisms remain unclear, particularly for methanotrophs. In the present study, we used a model strain of the gammaproteobacterial methanotroph, *Methylococcus capsulatus* strain Bath, to examine the characteristics of its usage of soluble and insoluble REEs.

We initially investigated whether various REEs affected the expression of the two MDHs in *M. capsulatus*. *M. capsulatus* was cultured in vial bottles with a capacity of 125‍ ‍mL, which were filled with 24‍ ‍mL of HEPES (4-[2-hydroxyethyl]-1-piperazineethanesulfonic acid)-buffered inorganic basal medium (pH 7.0), with a slight modification to the previously reported method (Kato *et al.*, 2020). CuCl_2_ and CaCl_2_ were omitted from the trace element solution and were separately added after autoclaving. Bottles were sealed with butyl rubber stoppers and aluminum seals before autoclaving. Methanol (20‍ ‍mM), CuCl_2_ (10‍ ‍μM), and CaCl_2_ or REE chlorides (Sc, Y, La, cerium [Ce], neodymium [Nd], or dysprosium [Dy]) (20‍ ‍μM) were supplemented from filter-sterilized stock solutions after autoclaving. Sc and Y were used as non-lanthanide REEs. La and Ce were selected because they have been frequently used as representatives of light lanthanide and are highly abundant in the environment (Elderfield *et al.*, 1990). Nd and Dy are representatives of light and heavy lanthanides, respectively, and were selected because of their high industrial demands as magnet materials. Cultures were performed at 37°C with agitation (180‍ ‍rpm). Growth was monitored by measuring optical density at 600‍ ‍nm (OD_600_) using a DS-11 spectrophotometer (DeNovix). After an 18-h incubation to reach the mid-log phase (OD_600_ of 0.1~0.2), cells were collected and treated with lysozyme solution (1‍ ‍mg mL^–1^) at room temperature for 5‍ ‍min. Total RNA was isolated using ISOGEN II reagent (Nippon Gene) combined with a bead-beating method, as previously described (Kato *et al.*, 2014). RNA was purified using an RNeasy Mini kit (Qiagen) with a DNase treatment (RNase-free DNase set; Qiagen) as described in the manufacturer’s instructions. The concentration of purified RNA was quantified using the Qubit 2.0 fluorometer (Thermo Fisher Scientific) with the Qubit RNA HS Assay Kit (Thermo Fisher Scientific) according to the manufacturer’s instructions. The primers used for quantitative RT-PCR targeting *mxaF* (Bath-mxaF-725f; 5′-ACA AGG ACA ACC CGC ATT AC-3′, Bath-mxaF-922r; 5′-TGG TCA TGG TCC ATT TGT TG-3′), *xoxF* (Bath-xoxF-108f; 5′-TCC GAA GAA CTT TGC GAC TT-3′, Bath-xoxF-308r; 5′-GCG TAG ACC TTG TGG GGA TA-3′), and *mopB* (Bath-mopB-57f; 5′-ACA GGC CGA AGA GAC TTT CA-3′, Bath-mopB-246r; 5′-GGT GGT GTT GCC TTC GTA AT-3′) were designed using Primer3 software (Untergasser *et al.*, 2012). A quantitative gene expression ana­lysis was performed by one-step real-time quantitative RT-PCR (qRT-PCR) using the LightCycler 96 Instrument (Roche Life Science) using RNA-direct SYBR Green Realtime PCR Master Mix (Toyobo) as previously described (Kato *et al.*, 2014). At least three biological replicates were subjected to a qRT-PCR ana­lysis. Standard curves were generated with serially diluted PCR products (10^3^–10^8^ copies mL^–1^) amplified with the respective primer sets. The expression of *mopB*, encoding a constantly expressed outer membrane protein (Karlsen *et al.*, 2003), was used to normalize expression values.

Supplementation with different REEs did not significantly affect the growth of *M. capsulatus*. In cultures supplemented with 20‍ ‍μM CeCl_3_ or LaCl_3_, the expression of *xoxF* was more than 10-fold higher than that in the control culture supplemented with 20‍ ‍μM CaCl_2_, whereas the expression of *mxaF* was 58- to 101-fold lower (Fig. 1A). Only a 3-fold increase in the expression of *xoxF* was observed in the culture supplemented with NdCl_3_, while that of *mxaF* remained unchanged. Furthermore, the expression of both *xoxF* and *mxaF* was not significantly affected in the cultures supplemented with non-lanthanide REEs (ScCl_3_ and YCl_3_) and heavy lanthanides (DyCl_3_). In many other methylo- and methanotrophs, the lanthanide switch was only induced by light lanthanides (typically La to Nd) (Vu *et al.*, 2016; Gu and Semrau, 2017). These results clearly showed that *M. capsulatus* has the lanthanide switch, similar to other methylo- and methanotrophs.

The concentration dependence of the lanthanide switch in *M. capsulatus* was then investigated. CeCl_3_ was used as a representative of light lanthanides that induce the lanthanide switch in *M. capsulatus*. *M. capsulatus* was cultured in the same inorganic medium as that described above, except for supplementation with different concentrations of CeCl_3_. RNA was extracted from mid-log phase cells and the expression of the *xoxF* and *mxaF* genes was quantified by qRT-PCR, as described above (Fig. 1B). The lanthanide switch was clearly observed at CeCl_3_ concentrations of 0.03‍ ‍μM and higher; *xoxF* was up-regulated by 14- to 21-fold and *mxaF* was down-regulated by 37- to 189-fold. Even under 0.01‍ ‍μM of CeCl_3_, the expression of* xoxF* and *mxaF* showed a 7-fold increase and 3-fold decrease, respectively. Previous studies reported that lanthanide switches in methanotrophs required submicromolar to micromolar orders of lanthanides to function (Chu and Lidstrom, 2016; Gu and Semrau, 2017; Zheng *et al.*, 2018). These findings indicate that the lanthanide switch in *M. capsulatus* was functional under a low lanthanide concentration.

The capability of *M. capsulatus* to utilize insoluble lanthanides was then assessed. *M. capsulatus* was cultured in inorganic medium supplemented with 1‍ ‍g‍ ‍L^–1^ cerium oxide (CeO_2_) instead of soluble Ca or REE chlorides. The concentration of the Ce ion was assessed using an inductivity coupled plasma optical emission spectrometer (ICP-OES, ULTIMA2; Horiba). RNA extraction and qRT-PCR were conducted as described above. After CeO_2_ was suspended in uninoculated inorganic medium and incubated for 4 days, the concentration of the Ce ion in the filtered supernatant was below the detection limit, suggesting that CeO_2_ was insoluble under the conditions used in the present study. The expression of *xoxF* and *mxaF* was up-regulated by 5-fold and down-regulated by 23-fold, respectively, following supplementation with CeO_2_ (Fig. 1B). The concentration of the Ce ion in the spent medium of the CeO_2_-supplemented *M. capsulatus* culture was 13.9±0.3 nM, which was sufficient to induce the lanthanide switch in *M. capsulatus*. The CeO_2_-dissolving activity of the cell-free spent medium of *M. capsulatus* grown under lanthanide-free conditions was also evaluated. After CeO_2_ was suspended in the spent medium and incubated for 4 days, 25.8±1.2 nM of the Ce ion was detected, suggesting that *M. capsulatus* secretes chemical compound(s) that dissolve insoluble lanthanide oxides.

The production of lanthanide-chelating compounds was examined using the Chrome Azurol Sulfonate (CAS) assay as previously described (Patel *et al.*, 2018) with minor modifications. In brief, a working solution containing CAS (final concentration of 18.75‍ ‍μM) and hexadecyltrimethylammonium bromide (final concentration of 50‍ ‍μM) and buffered with 1.5‍ ‍mM each of 2-(N-morpholino)ethanesulfonic acid (MES) and 4-(2-hydroxyethyl)-1-piperazineethanesulfonic acid was prepared. Fifteen microliters of 0.25‍ ‍mM CeCl_3_ (final concentration of 18.75‍ ‍μM) and 40‍ ‍μL of concentrated spent medium were then applied to 145‍ ‍μL of the buffered working solution. The mixture was incubated at room temperature with agitation (180‍ ‍rpm) for 5‍ ‍mins. An absorbance scan was performed with a range of 350‍ ‍nm to 750‍ ‍nm in 1-‍nm intervals using the Tristar 5 Multimode Reader (Berthold Technologies). *M. capsulatus* was cultured in the inorganic medium supplemented with different concentrations of CeCl_3_. The spent medium was collected in the late log phase and subjected to reversed-phase chromatography in order to concentrate it by approximately 100-fold. The concentrated spent medium was analyzed using the CAS assay. In the case of the control sample (uninoculated medium) and the spent medium supplemented with 0.1‍ ‍μM or more of CeCl_3_, an absorbance peak at 630‍ ‍nm, which is characteristic of the CAS-Ce complex, was observed (Fig. 2). In contrast, the absorbance peak at 630‍ ‍nm disappeared when the spent medium was obtained from cultures supplemented with 0.03‍ ‍μM or less of CeCl_3_. In addition to chelating compounds, the decrease in CAS-Ce complexes was affected by non-specific metal adsorbents. However, the production of these adsorbents was not expected to be significantly affected by culture conditions. These results indicate that *M. capsulatus* produce Ce-chelating compound(s) only under lanthanide-deficient conditions.

A transcriptome ana­lysis based on RNA sequencing was performed to identify the genes responsible for synthesizing chelating compounds. RNA sequencing was performed using the DNBSEQ-G400 sequencer by Bioengineering Lab. Raw reads were trimmed by Trimmomatic v0.39 (Bolger *et al.*, 2014) and mapped to the genome of *M. capsulatus* Bath (GCA_000008325) using BWA v0.7.17 (Li and Durbin, 2009). Gene expression levels were calculated as transcripts per million (TPM) using StringTie v2.2.1 (Pertea *et al.*, 2016) and then normalized to the average of the ribosomal protein level of each sample. Transcriptomics data were deposited in NODE (http://www.biosino.org/node) with the accession number PRJDB16341. Genes with expression levels that were higher or lower (>2-fold expression change and p<0.05) in the Ce-supplemented culture (supplemented with 0.1‍ ‍μM CeCl_3_) than in the control culture (supplemented with 0.1‍ ‍μM CaCl_2_) are listed in supplementary Table S1. As shown by the CAS assay, the genes required for the synthesis of lanthanide chelator(s) were expected to be down-regulated in the Ce-supplemented culture. The genes for MxaFI-MDH (MCA0779 and 0782), its putative regulatory genes (MCA0776-0778), and its accessory genes (MCA0780-0781 and 783-789) were shown to be down-regulated under Ce-supplemented conditions. Furthermore, genes for formate dehydrogenase (FDH) (MCA2576-2577) and its downstream ABC transporter (MCA2578-2580) were down-regulated under Ce-supplemented conditions. The down-regulation of FDH following the addition of lanthanide was previously reported in the methylotroph *Methylobacterium extorquens* AM1, likely to remodel its metabolic flux in order to reduce formate oxidation and balance NAD(P)H production (Good *et al.*, 2019). The 5 other down-regulated genes were annotated as proteins with no clear presumed function (Table S1). Among them, MCA1883, annotated as a non-ribosomal peptide synthetase, was a candidate for the synthesis of chelator compounds. An ana­lysis using antiSMASH, which predicts secondary metabolite biosynthetic gene clusters (Blin *et al.*, 2023), revealed that MCA1883 is part of a large biosynthetic gene cluster consisting of MCA1865 to 1899 (supplementary Fig. S1). Clustered genes were analyzed using the structure-function prediction program Phyre2 (Kelley *et al.*, 2015). MCA1883 showed homology (27% identity) with enterobactin synthase component F, a gene responsible for the synthesis of the iron chelator enterobactin (Raymond *et al.*, 2003). The construction of gene-disrupting mutants and the purification of putative chelator compounds are ongoing in our laboratory and will provide insights into the mole­cular mechanisms underlying lanthanide acquisition, particularly the production of lanthanide-chelating molecule(s) by *M. capsulatus*.

In conclusion, we showed that *M. capsulatus* has the “lanthanide switch” as observed in other methylo- and methanotrophs. The lanthanide switch in *M. capsulatus* was induced by both soluble lanthanide chlorides and insoluble oxides. Furthermore, the spent medium of *M. capsulatus* exhibited activity to dissolve Ce oxide and contained Ce-chelating chemical(s). The transcriptome ana­lysis showed that the genes required for the synthesis of an enterobactin-like metal chelator were up-regulated under lanthanide-deficient conditions. These results indicate that *M. capsulatus* produces lanthanide-chelating compounds to sequester insoluble lanthanide oxides.

## Citation

Xie, R., Takashino, M., Igarashi, K., Kitagawa, W., and Kato, S. (2023) Transcriptional Regulation of Methanol Dehydrogenases in the Methanotrophic Bacterium *Methylococcus capsulatus* Bath by Soluble and Insoluble Lanthanides. *Microbes Environ ***38**: ME23065.

https://doi.org/10.1264/jsme2.ME23065

## Supplementary Material

Supplementary Material

## Figures and Tables

**Fig. 1.**
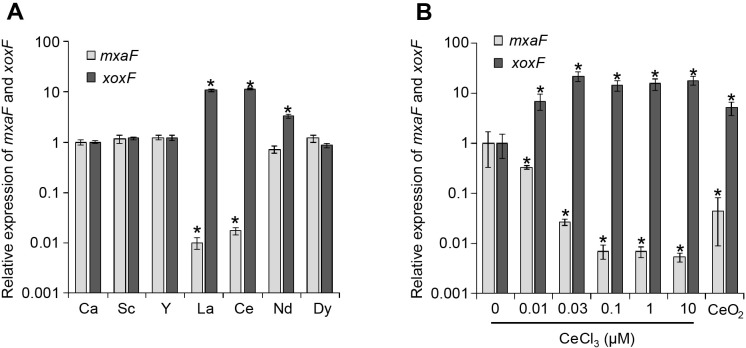
Expression of *mxaF* and *xoxF* in *Methylococcus capsulatus* in the presence of CaCl_2_ or different REE chlorides (A) and different concentrations of Ce chloride or insoluble CeO_2_ (1‍ ‍g‍ ‍L^–1^) (B). The expression of *mxaF* and *xoxF* in mid-log phase cells was assessed by qRT-PCR. Expression was normalized by the expression of the housekeeping gene *mopB*. Data are presented as the means of triplicate experiments, and error bars represent standard deviations. Asterisks represent a significant difference (>2-fold change, *P*<0.05) from CaCl_2_-supplemented (A) or no lanthanide (B) control cultures. Data were statistically analyzed by the Student’s *t*-test.

**Fig. 2.**
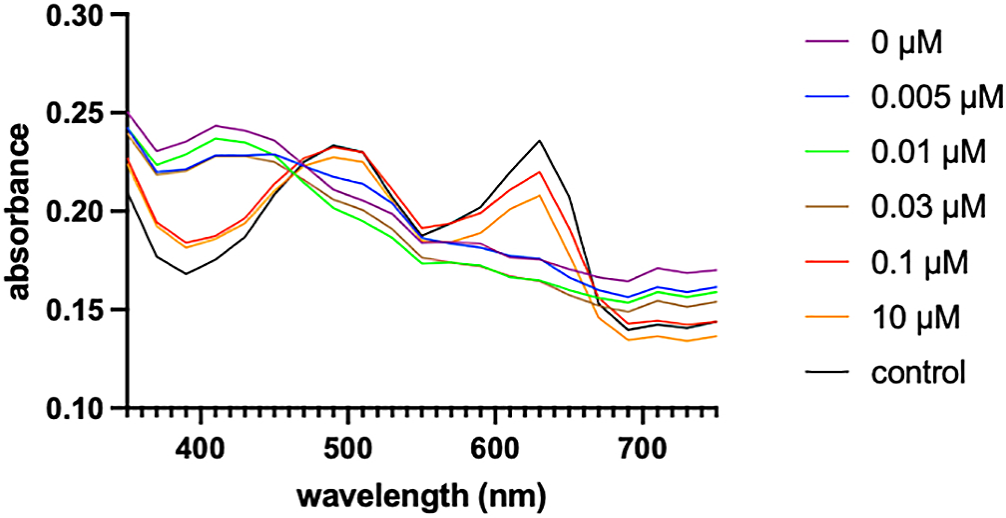
Detection of Ce-chelating compound(s) by the Chrome Azurol Sulfonate (CAS) assay. Buffered solution containing CAS, hexadecyltrimethylammonium bromide, and CeCl_3_ was mixed with an uninoculated medium (control) or the concentrated spent medium of *Methylococcus capsulatus* cultures with different concentrations of CeCl_3_ (0 to 10‍ ‍μM). After the incubation, absorbance was scanned with a range of 350‍ ‍nm to 750‍ ‍nm. The decrease observed in the absorbance peak at 630‍ ‍nm due to the CAS-Ce complex indicates the presence of Ce-chelating compounds.
