# Supplementary material for: Transcriptional Regulation of Methanol Dehydrogenases in the Methanotrophic Bacterium Methylococcus capsulatus Bath by Soluble and Insoluble Lanthanides
Source: Microbes Environ. 2023 Dec 12;38(4):ME23065. doi: 10.1264/jsme2.ME23065 (PMC10728633; doi:10.1264/jsme2.ME23065)
Supplement: Supplementary file 1 — Supplementary Material [file 38_23065_s1.pdf]

Supplementary Information

Utilization of insoluble lanthanide oxides by a methanotrophic bacterium *Methylococcus capsulatus* Bath

Xie Ruoyun[1,2], Motoko Takashino[2], Kensuke Igarashi[2], Wataru Kitagawa[1,2], Souichiro Kato[1,2,*]

[1]*Division of Applied Bioscience, Graduate School of Agriculture, Hokkaido University, Kita-9 Nishi-9, Kita-ku, Sapporo 060-8589, Japan*; [2]*Bioproduction Research Institute, National Institute of Advanced Industrial Science and Technology, 2-17-2-1 Tsukisamu-Higashi, Toyohira-ku, Sapporo 062-8517, Japan.*

*Corresponding author: E-mail: s.katou@aist.go.jp; Tel.: (+81) 11 857 8968; Fax: (+81) 11 857 8915

Figs. S1

Table S1

Fig. S1. A putative gene cluster for an enterobactin-like metal chelator. The gene cluster was found by antiSMASH that predicts secondary metabolite biosynthetic gene clusters. The genes predicted to be involved in biosynthesis are highlighted and numbered. The gene IDs, functions predicted by antiSMASH or Phyre2, identities, and coverage for the putative biosynthesis genes are listed in the table. NPRS; non-ribosomal peptide synthetase.

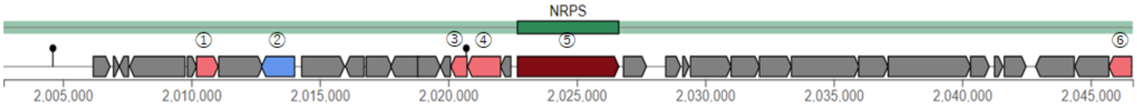

No.	Gene ID	gene products predicted in antiSMASH	homology identified in Phyre2	identities	coverage
**1**	MCA1870	ribD C-terminal domain protein	oxidoreductase	23%	77%
**2**	MCA1872	C4-dicarboxylate transporter	proton/glutamate symporter	30%	93%
**3**	MCA1879	nicotinate (nicotinamide) nucleotide adenylyltransferase	adenylyltransferase	49%	98%
**4**	MCA1880	gamma-glutamyl phosphate reductase	gamma-glutamyl phosphate reductase	63%	96%
**5**	MCA1883	non-ribosomal peptide synthetase	enterobactin synthase component f	27%	98%
**6**	MCA1899	cysteine synthase B	Tryptophan synthase beta subunit-like PLP-dependent enzymes	65%	98%

Table S1. Summary of the transcriptome analysis. Genes up- or down-regulated (>2-fold expression change and $p<0.05$) in the Ce-supplemented culture (supplemented with 0.1 μM $CeCl_3$) compared to the control culture (supplemented with 0.1 μM $CaCl_2$) were listed.

Predicted functions	Gene ID	Gene Name	log_2 fold change	*p* value
**Up-regulated genes in the Ce-supplemented condition**				
Xox-type methanol dehydrogenase	MCA0299	*xoxF*	2.84	2.63E-03
	MCA0300		1.96	1.39E-03
unknown functions	MCA0898	-	1.33	2.96E-05
	MCA0899	-	1.46	3.84E-03
	MCA0900	-	1.38	9.20E-03
	MCA1183	*cysT*	1.21	4.47E-04
	MCA1886	-	1.52	2.13E-02
	MCA2279	*cpxR*	1.42	1.50E-03
	MCA2280	-	1.91	3.75E-03
	MCA2507	-	1.24	1.03E-02
**Down-regulated genes in the Ce-supplemented condition**				
Mxa-type methanol degydrogenase and its accessory genes	MCA0776	-	-1.55	5.13E-03
	MCA0777	-	-2.14	1.88E-03
	MCA0778	-	-2.91	2.19E-03
	MCA0779	*mxaF*	-7.52	3.53E-04
	MCA0780	*mxaJ*	-7.47	3.32E-03
	MCA0781	-	-7.16	2.01E-03
	MCA0782	*mxaI*	-6.93	7.80E-04
	MCA0783	*moxR*	-6.46	2.45E-03
	MCA0784	-	-6.53	8.50E-03
	MCA0785	*mxaA*	-5.32	4.06E-03
	MCA0786	*mxaC*	-5.85	6.97E-03
	MCA0787	*mxaK*	-4.86	5.75E-03
	MCA0788	*mxaL*	-4.80	8.99E-03

	MCA0789	*mxaD*	-4.82	8.46E-03
formate dehydrogenases	MCA2576	-	-2.07	7.17E-03
	MCA2577	-	-2.75	4.95E-03
ABC transporters	MCA2578	-	-3.10	6.57E-03
	MCA2579	-	-1.79	1.70E-05
	MCA2580	-	-2.87	1.36E-03
unknown functions	MCA0715	-	-2.18	3.76E-03
	MCA0791	-	-1.31	1.02E-02
	MCA1883	-	-1.29	3.90E-06
	MCA2575	-	-1.66	4.04E-03
	MCA2618	-	-1.24	3.51E-04
